# Genomic analysis of *Leptospira interrogans* serovar Paidjan and Dadas isolates from carrier dogs and comparative genomic analysis to detect genes under positive selection

**DOI:** 10.1186/s12864-019-5562-z

**Published:** 2019-03-04

**Authors:** Alongkorn Kurilung, Chantisa Keeratipusana, Prapat Suriyaphol, David J. Hampson, Nuvee Prapasarakul

**Affiliations:** 1 Department of Microbiology, Faculty of Veterinary Science, Chulalongkorn University, Bangkok, Thailand; 2 Bioinformatics and Data Management for Research Unit, Office for Research and Development, Faculty of Medicine Siriraj Hospital, Mahidol University, Bangkok, Thailand; 3 Department of Infectious Diseases and Public Health, College of Veterinary Medicine and Life Sciences, City University of Hong Kong, Kowloon Tong, Hong Kong SAR; 4 Diagnosis and Monitoring of Animal Pathogens Research Unit, Department of Microbiology, Faculty of Veterinary Science, Chulalongkorn University, Bangkok, Thailand

**Keywords:** Genome, *Leptospira interrogans*, Serovar Paidjan, Serovar Dadas, Dog, Positive selection

## Abstract

**Background:**

Leptospirosis is an emerging infectious disease worldwide that can cause high morbidity and mortality rates in humans and animals. The causative spirochetes have reservoirs in mammalian hosts, but there has been limited analysis of the genomes of isolates recovered from animals. The aims of this study were to characterize genomic features of two *Leptospira interrogans* strains recently isolated from asymptomatic dogs in Thailand (strains CUDO5 and CDUO8), and to perform comparative genome analyses with other strains. Molecular adaptive evolution in *L. interrogans* as signaled by positive selection also was analyzed.

**Results:**

Whole genome sequence analysis revealed that strains CUDO5 and CUDO8 had genome sizes of approximately 4.9 Mbp with 35.1% GC contents. Using monoclonal antibodies, strains CUDO5 and CUDO8 were identified as serovars Paidjan and Dadas, respectively. These strains harbored genes known to be associated with acute and chronic infections. Using Single Nucleotide Polymorphisms phylogeny (SNPs) with 97 *L. interrogans* strains, CUDO5 and CUDO8 had closest genetic relatedness with each other. Nevertheless, the serovar determinant region (*rfb* locus) showed variations in the genes encoding sugar biosynthesis. Amongst 13 representative *L. interrogans* strains examined for molecular adaptive evolution through positive selection under the site-model of Phylogenetic Analysis of Maximum Likelihood, genes responsible for iron acquisition (*tlyA* and *hbpA*), motility (*fliN2*, *flgK*, and *flhB*) and thermal adaptation (*lpxD1*) were under increased selective pressure.

**Conclusions:**

*L. interrogans* serovar Paidjan strain CUDO5 and serovar Dadas strain CUDO8 had close genetic relatedness as analyzed by SNPs phylogeny. They contained genes with established roles in acute and chronic leptospirosis. The *rfb* locus in both serovars showed gene variation associated with sugar biosynthesis. Positive selection analysis indicated that genes encoding factors involved in motility, temperature adaptation, and iron acquisition were under strong positive selection in *L. interrogans*. These may be associated with adaptation in the early stages of infection.

**Electronic supplementary material:**

The online version of this article (10.1186/s12864-019-5562-z) contains supplementary material, which is available to authorized users.

## Background

Gram-negative spirochetes of the genus *Leptospira* cause the disease Leptospirosis, which is one of the most important zoonotic infectious conditions worldwide, including in Thailand [1, 2]. Pathogenic *Leptospira* strains can infect most mammals and produce a wide range of clinical manifestations varying from acute life-threatening disease to chronic asymptomatic forms [3]. In humans, leptospirosis is estimated to have a worldwide annual average morbidity of 1.03 million cases with a mortality of 58,900 cases, and these figures are poised to increase due to climate change [4]. Asymptomatic animals that are carrying leptospires in the convoluted tubules of the kidney play an important role in disease maintenance and in transmission via infected urine [1].

Based on DNA-DNA hybridization and 16S rRNA phylogeny, *Leptospira* can be classified into more than 22 species located within three groups: the pathogenic, intermediate and non-pathogenic groups. In addition, more than 300 serovars have been described based on antigenic diversity in the lipopolysaccharide (LPS) structure on the cell surface. These different serovars can be identified using the cross-agglutination absorption test (CAAT) [3].

Pathogenic *L. interrogans* serovar Lai strain 56601 was the first leptospire to be subjected to genome sequencing, and the data obtained revealed the unique physiological characteristics and virulence factors of this strain [5]. Subsequently, the genomes of a number of other both pathogenic and non-pathogenic *Leptospira* strains have been subjected to next-generation sequencing (NGS) to investigate the genetic basis of pathogenesis [6]. Using comparative genomic analysis, pathogenic markers were defined based on gene gain and loss events, and genome rearrangements were identified [6–8]. An aspect that has not been explored for *Leptospira* is measurement of selective pressure, inferred by non-synonymous to synonymous substitution rate in genes in conserved genome regions, signaling that the genes are under positive selection: in other species these adaptations have been shown to have an important role in species diversification and adaptation in different ecological niches and hosts [9].

In pathogenic bacteria, positive selection is an evolutionary driving force arising by mutations in protein-coding genes that are associated with bacterial adaptation to changes in their environment or host species [10]. Advantageous point mutations enhance bacterial physiological fitness or virulence factor profiles appropriate to survival under various conditions [11]. Genome-wide analyses of positive selection associated with increased rates of point mutations in specific genes has been reported for several bacteria, including *Bacteroides fragilis* [12]*, Escherichia coli* [13, 14], *Mycobacterium tuberculosis* [15]*, Salmonella* serotypes [16] and *Pasteurella multocida* [17]. To date reports of positive selection in *Leptospira* remain scarce, and there is still a lack of genomic information for *Leptospira* isolates recovered from asymptomatic animals.

In the present study, we determined the serotypes and then sequenced the genomes of two *L. interrogans* isolates that were recently recovered from the urine of asymptomatic Thai dogs. We then undertook comparative analysis with these genomes and 22 other representatives of validly published *Leptospira* genomes obtained from the NCBI database. The Thai *L. interrogans* strains were subjected to genomic characterization and phylogenetic analysis to examine evolutionary relationships. Within-species, comparative genome analysis was performed with 97 strains of *L. interrogans* to obtain insight into the pan-genome structure. Strain-specific genes of the two Thai strains that were associated with specific functions including virulence also were identified. Furthermore, a genome-wide analysis for positive selection was undertaken on the core genes of *L. interrogans* to determine genes involved in adaptive evolution. The results provide insight into the *Leptospira* genome and contribute to a more comprehensive understanding of factors involved in molecular pathogenesis and adaptive evolution in pathogenic *Leptospira*.

## Results

### Genome characteristics of the two Thai *L. interrogans* strains

**Fig. 1:**
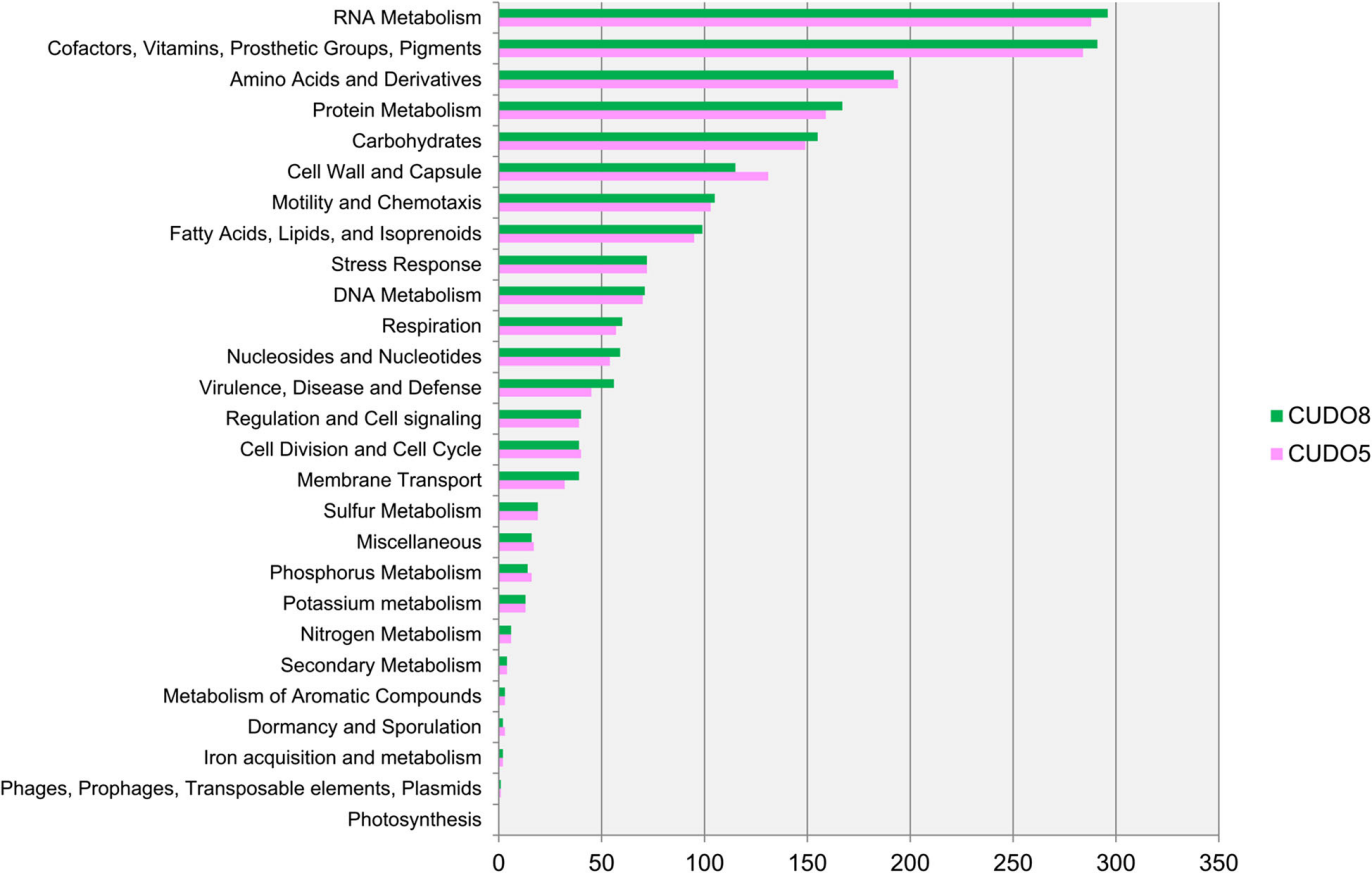
RAST subsystem annotation in *L. interrogans* serovar Paidjan strain CUDO5 (pink bar) and serovar Dadas strain CUDO8 (green bar). The functional subsystem category with the largest number of annotations is RNA metabolism, following by Cofactors, vitamins, prosthetic groups, pigments, and Amino acids and derivatives, respectively

The basic genome characteristics of all analyzed *Leptospira* strains are summarized in Table 1. For *L. interrogans* strain CUDO5, the A5-Miseq de novo genome assembler generated 163 scaffolds with 124.47 times genome coverage and an N50 of 111,967 bp: the genome size was estimated at 4.94 Mbp with a 35.1% GC content. For strain CUDO8, 83 scaffolds were obtained with 109.34 times genome coverage and an N50 of 165,528 bp, with a genome size of approximately 4.91 Mbp and a 35.1% GC content. PROKKA annotation showed that strains CUDO5 and CUDO8 had 4040 and 4013 predicted coding sequences (CDSs), respectively. Both strains only had one copy of each of the three ribosomal RNA genes (5S, 16S and 23S). RAST categorized the functions of the CDSs in strains CUDO5 and CUDO8 into 323 and 320 sub-systems containing 1896 and 1936 CDSs, respectively. Most CDSs in strains CUDO5 and CUDO8 respectively related to RNA metabolism (288/1896 and 296/1936), cofactors, vitamins, prosthetic groups, pigments (284/1896 and 291/1936), amino acids and derivatives (194/1896 and 192/1936), protein metabolism (159/1896 and 167/1936), carbohydrates (149/1896 and 155/1936), cell wall and capsule (131/1896 and 115/1936), and motility and chemotaxis (103/1896 and 105/1936) (Fig. 1). By comparison within and between different strains, the number of CDSs in *Leptospira* species varied from 3600 to 4342 by PROKKA annotation. In RAST analysis *L. santarosai* and *L. interrogans* serovar Lai had the lowest and highest number of CDSs, at 1513 and 1952, respectively (Additional file 1: Table S1).

**Table 1:** Representative *Leptospira* species and strains used in the study

Species	Strain	Serovar	Species of origin	Genome status	Size (Mbp)	Number of CDS	GC%	Accession number	Abbreviated name in this study
*L. interrogans*	CUDO5	Paidjan	Dog	Draft	4.94	4040	35.1	NKYH02000001.1	CUDO5
*L. interrogans*	CUDO8	Dadas	Dog	Draft	4.91	4013	35.1	NKYG00000001.1	CUDO8
*L. interrogans*	56601	Lai	Human	Complete	4.62	3776	35.0	NC_004342.2	LA
*L. interrogans*	Fiocruz L1–130	Copenhageni	Human	Complete	4.62	3680	35.0	NC_005823.1	LIC
*L. interrogans*	LP101	Autumnalis	Human	Draft	5.03	4111	35.2	NZ_AHNF02000127.1	AUT
*L. interrogans*	LT1649	Djasiman	Human	Draft	4.67	3740	34.9	NZ_AFMB02000229.1	DJAS
*L. interrogans*	PigK151	Batislava	Pig	Complete	4.72	3775	35.0	NZ_CP011410.1	BTL
*L. interrogans*	L1111	Bataviae	Human	Draft	4.85	3984	35.1	NZ_AHND02000082.1	BTV
*L. interrogans*	Fiocruz LV133	Canicola	Human	Draft	4.71	3810	35.0	NZ_AKWU02000089.1	CAN
*L. interrogans*	UI 08368	Grippotyphosa	Human	Draft	4.87	3910	35.0	NZ_AHNJ02000078.1	GRP
*L. interrogans*	AKRFB	Pomona	Cattle	Draft	4.62	3724	35.0	NZ_LUHH01000024.1	PMN
*L. interrogans*	R168	Pyrogenes	Human	Draft	4.86	3979	35.2	NZ_AHNH02000099.1	PYR
*L. interrogans*	Norma	Hardjo	Cattle	Complete	4.76	3876	35.0	NZ_CP012603.1	HJ
*L. kirschneri*	3522 CT	Cynopteri	Bat	Draft	4.40	3605	35.9	NZ_AHMN02000024.1	CYN
*L. noguchii*	CZ 214	Panama	Opossum	Draft	4.71	4048	35.5	NZ_AKWY02000035.1	NOGU
*L. borgpetersenii*	L550	Hardjo-bovis	Human	Complete	3.89	3606	40.2	NC_008508.1	LBL
*L. weilii*	LNT1234	undetermined	Human	Draft	4.26	3876	40.4	NZ_AHNC02000083.1	WEIL
*L. alexanderi*	L 60	Manhao 3	Human	Draft	4.22	3994	40.2	NZ_AHMT02000065.1	ALEX
*L. alstonii*	80–412	Pingchang	Frog	Draft	4.59	4139	42.4	NZ_CP015217.1	ALS
*L. santarosai*	1342 K	Shermani	Human	Draft	4.03	3600	41.8	NZ_CP006694.1	SANTA
*L. kmetyi*	Bejo-Iso9	Malaysia	Soil	Draft	4.41	3994	44.8	NZ_AHMP02000004.1	KMET
*L. licerasiae*	VAR010	Varillal	Human	Draft	4.21	3865	41.1	NZ_AHOO02000014.1	LINC
*L. wolffii*	Khorat-H2	Khorat	Human	Draft	4.40	4036	45.6	NZ_AKWX02000023.1	WOLF
*L. biflexa*	Patoc	Patoc I (Paris)	Water	Complete	3.95	3687	38.9	NC_010602.1	LBF

### Prophages, CRISPR/Cas and putative virulence factors

According to PHASTER analysis, no intact bacteriophage (phage) was identified in strains CUDO5 or CUDO8. However, incomplete phages with sizes ranging from 6.1 to 11.4 Kbp were found in both strains: these included PHAGE_Synech_ACG_2014b_NC_027130, PHAGE_Synech_ACG_2014f_NC_026927, PHAGE_Psychr_pOW20_A_NC_020841 and PHAGE_Sphing_PAU_NC_019521. None of these predicted phages contained any sequences associated with known pathogenicity islands or antimicrobial resistance genes. Most of the *Leptospira* species and strains contained incomplete phages with sizes ranging from 4.1 to 13.8 Kbp, but the only one shared was PHAGE_Sphing_PAU_NC_019521, which was found in 11 of the 13 *L. interrogans* strains (Additional file 1: Table S2).

**Fig. 2:**
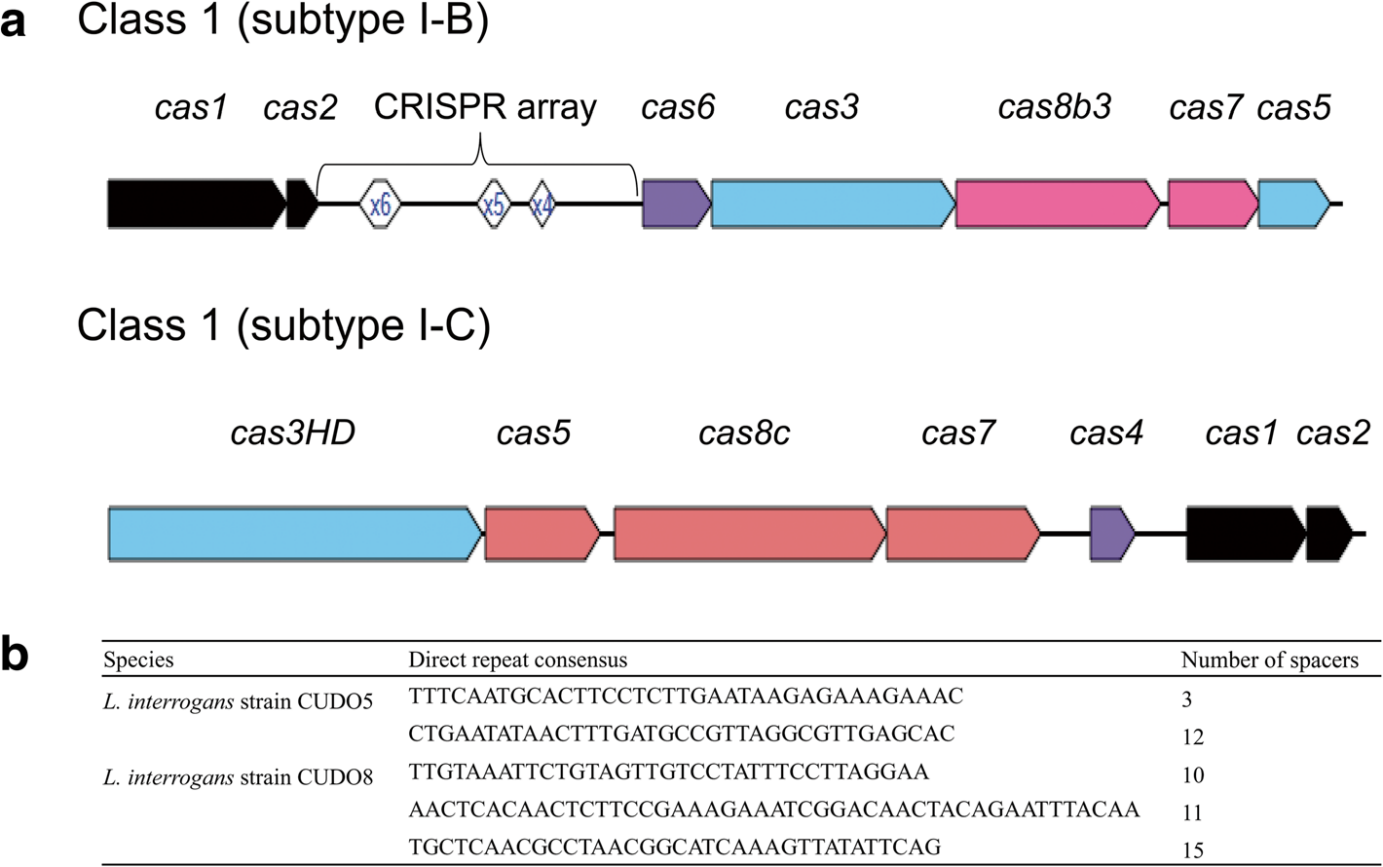
CRISPR-Cas gene organization in *L. interrogans* serovar Paidjan strain CUDO5 and serovar Dadas strain CUDO8. Strains CUDO5 and CUDO8 contained CRISPR1 subtypes I-B and I-C. CRISPR1 subtype I-B is a complete locus flanked with a CRISPR array which harbors specific direct repeats (DRs) and spacers (panel **a**), with different quantities based on the time of exogenous DNA exposure (panel **b**)

**Fig. 3:**
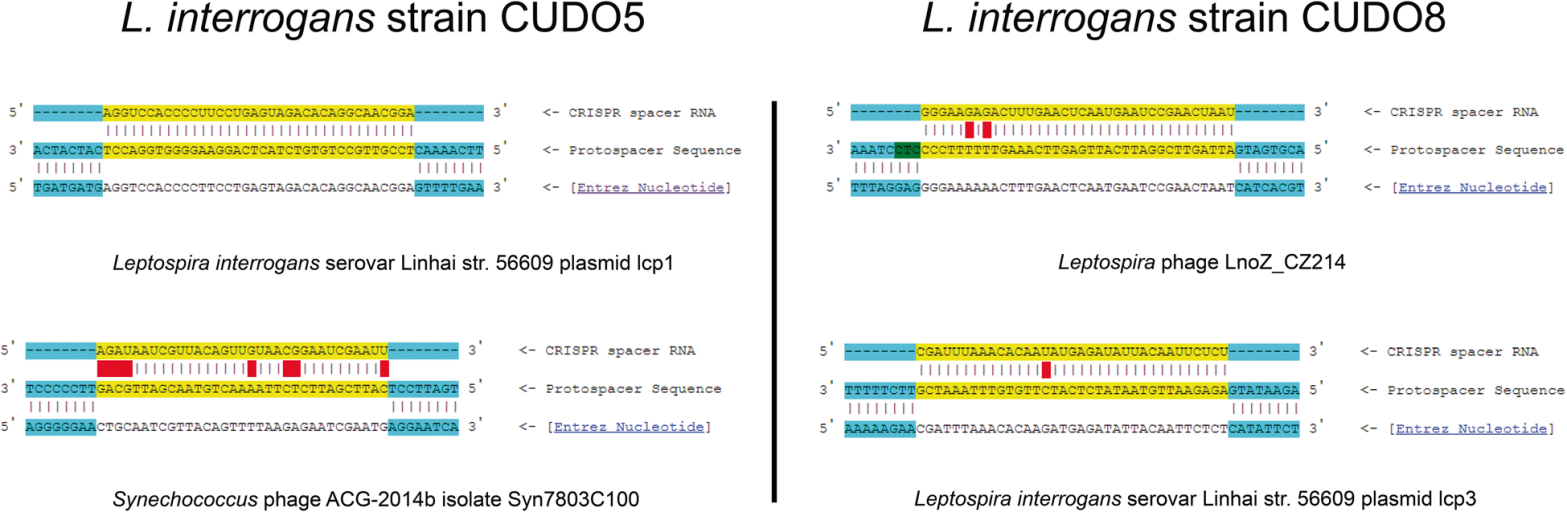
Graphical display of CRISPR RNA (crRNA) and target protospacer DNA in *L. interrogans* serovar Paidjan strain CUDO5 and serovar Dadas strain CUDO8. Spacer specific sequences are transcribed to crRNA to protect against phage and plasmid re-infection. The crRNA in strains CUDO5 and CUDO8 gave highest matching scores to a plasmid of *L. interrogans* serovar Linhai strain 56609. Colored yellow indicates the complementary matching between spacer and target protospacer sequence; red indicates the mismatching of spacer and target protospacer sequence and blue represents the flanking sequence between spacer and target protospacer

A CRISPR-Cas system was identified in strains CUDO5, CUDO8 and in some of the other strains using the CRISPRone web tool. Interestingly, non-pathogenic *L. biflexa* lacked this system. Direct repeats (DRs) were not identified in the genomes of *L. borgpetersenii*, *L. alstonii*, *L. kmetyi*, *L. lincerasiae* and *L. wolffi*; however, specific Cas proteins related to the CRISPR-Cas system were predicted in these *Leptospira* species. In this study, we followed the CRISPR-Cas system classification criteria that broadly categorize the CRISPR-Cas system into two classes (Class 1 and Class 2). The CRISPR-Cas system class 1 (CRISPR1) consists of three types (type I, III and IV), whilst the CRISPR-Cas system class 2 (CRISPR2) harbors two types (type II and V), with subdivision into 16 distinct subtypes based on different combinations of Cas protein [18]. Based on the presence of specific Cas proteins, several CRISPR1 subtypes were predicted in representative *Leptospira* species, including subtypes I-A, I-B, I-C and I-E (Additional file 1: Table S3). Among the *L. interrogans* strains, including CUDO5 and CUDO8, two CRISPR1 loci (subtype I-B and subtype I-C) were found (Fig. 2a). The CRISPR1 subtype I-B locus contained seven *cas* genes (*cas1*, *cas2*, *cas3*, *cas5*, *cas6*, *cas7* and *cas8b3*) flanked with different numbers of DRs and spacers, while the CRISPR1 subtype I-C was an orphan locus that harbored seven *cas* genes (*cas1*, *cas2*, *cas3HD*, *cas4, cas5*, *cas7* and *cas8c*) without a flanking CRISPR array region. The CRISPR array region of strain CUDO5 consisted of 4 DRs with nucleotide lengths from 36 to 39 bp and 15 spacers, whereas strain CUDO8 harbored 4 DRs with nucleotide lengths from 35 to 48 bp and had 36 spacers (Fig. 2b). Spacer sequences predicted from CRISPRone were used to find CRISPR RNA (crRNA) targeting specific foreign sequences using the CRISPRTarget web tool. The plasmid lcp1 of *L. interrogans* serovar Linhai strain 56609 and *Synechococcus* phage ACG-2014b gave maximum matching scores in strain CUDO5. On the other hand, *Leptospira* phage LnoZ_CZ214 and plasmid lcp3 of *L. interrogans* serovar Linhai strain 56609 showed maximum matching scores in strain CUDO8 (Fig. 3).

**Fig. 4:**
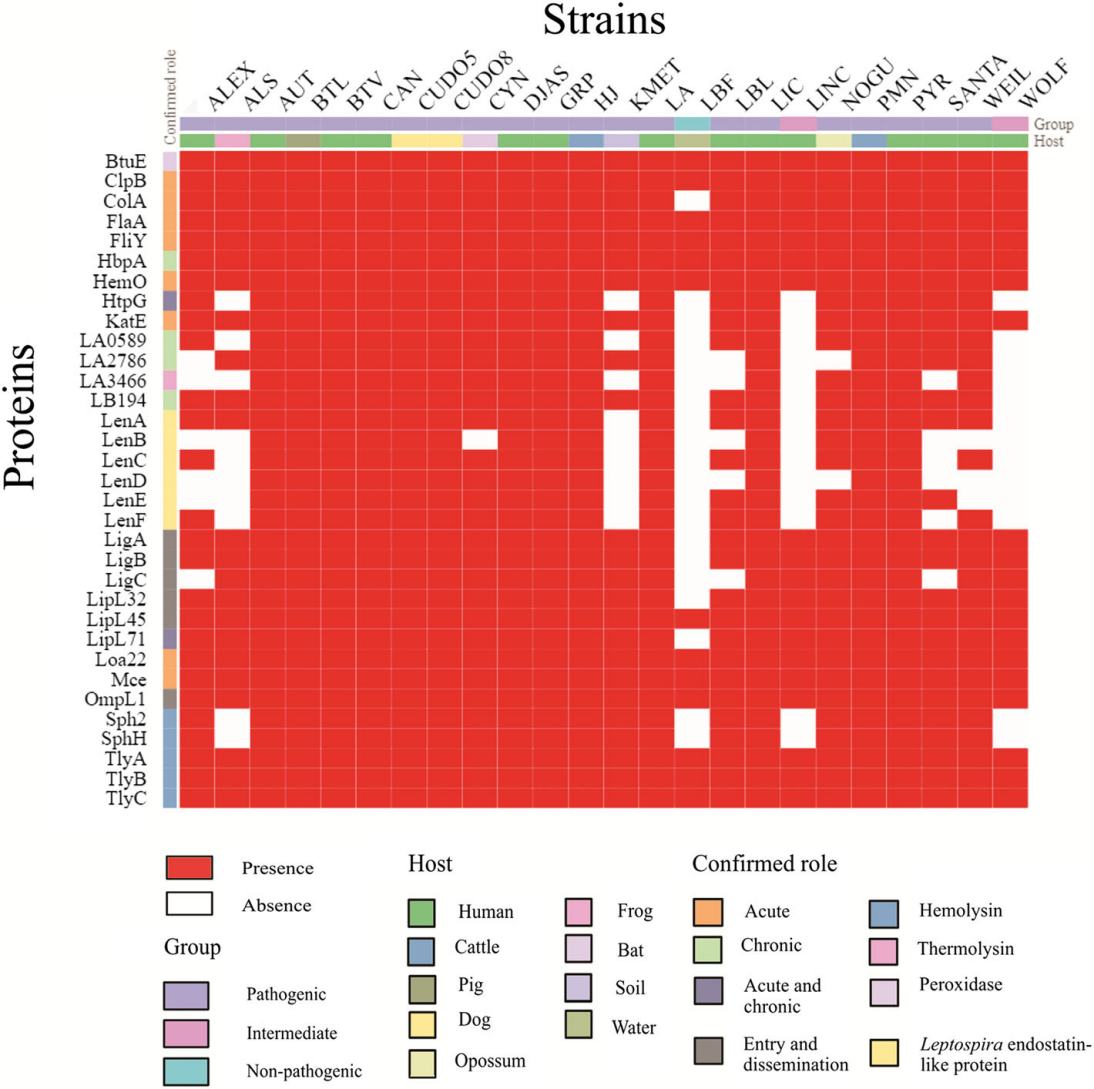
The distribution and conservation of 33 representative confirmed virulence genes in pathogenic, intermediate and non-pathogenic *Leptospira* species. Genes encoding for outer membrane protein (*loa22*), flagella motor switch protein (*fliY*), and hemolysins B and C (*tlyB* and *tlyC*) were conserved in all 24 *Leptospira* strains. Genes encoding was conserved only in pathogenic *Leptospira* strains. Strains CUDO5 and CUDO8 contained all of the 33 virulence genes

By searching in the VFDB database, the genomes of *L. interrogans* strains CUDO5 and CUDO8 were shown to encode 518 and 512 putative virulence factors, respectively. The predicted virulence genes were involved with motility, chemotaxis, toxins, adhesion molecules and iron uptake systems, amongst others (Additional file 1: Tables S5 and S6). The BLAST results for gene orthologous identification among representative *Leptospira* strains using amino acid sequences from 33 confirmed virulence factors showed that BtuE, ClpB, FlaA, FliY, HbpA, HemO, LipL45, Loa22, Mce, OmpL1, TlyA, TlyB and TlyC were distributed in all *Leptospira* strains. Genes encoding KatE, HtpG, ColA, Lig and Sph tended to be restricted to pathogenic *Leptospira* strains. Strains CUDO5 and CUDO8 were found to possess all core virulence-associated genes involved in acute and chronic leptospirosis infection (Fig. 4) (Additional file 1: Table S7).

### Serotyping, *rfb* locus, and lipid A and sialic acid biosynthesis encoding genes

In the current study, serotyping using pAbs and mAbs showed that *L. interrogans* strain CUDO5 belonged to serogroup Bataviae (pAbs titer = 1:640), with close similarity to *L. interrogans* serovar Paidjan (mAbs titer = 1:20480), whilst strain CUDO8 belonged to serogroup Grippotyphosa (pAbs titer > 1:1280) with antigenic similarity to *L. kirschneri* serovar Dadas (mAbs titer = 1:20480).

Analysis of the lipopolysacharide biosynthesis (*rfb*) locus from the whole genome sequence data demonstrated that serovar Paidjan (strain CUDO5) contained nucleotide sequences of approximately 116 Kbp, which was larger than for serovar Dadas (strain CUDO8) that harbored nucleotides of around 114 Kbp. These regions encoded 125 and 113 genes for serovar Paidjan and Dadas, respectively (Additional file 1: Tables S8 and S9). In agreement with a previous report [6], the upstream and downstream flanking genes in the *rfb* locus were conserved, with *petE* encoding copper-binding protein and *rpsF* encoding 30S ribosomal protein S6 being present in both serovars (Additional file 2: Figure S1). The complete gene cluster of dTDP-rhamnose biosynthesis (*rfb ABCD*) and genes related to rhamnose containing glycan (*rfbB* and *galE*) were found in these two *rfb* loci. Besides rhamnose biosynthesis genes, serovar Dadas contained additional genes involved with capsular heptose biosynthesis (*fcl*, *gmhA* and *rfbC*). Sialic acid biosynthesis genes including *neuA1* and *neuB2* were found within the *rfb* locus of serovar Paidjan. The gene encoding bacillosamine/legionaminic acid aminotransferase (*pglE*), which plays a role in protein glycosylation via the *N,N*′-diacetylbacillosamine biosynthesis pathway, was found in both serovars. Three aminotransferases belonging to the DegT family that are involved with LPS O-antigen side chain biosynthesis were identified in serovar Paidjan. Genes related to the Wzx/Wzy-dependent pathway (*wzx* and *wzy*) were not situated in the *rfb* locus of either strain, but were located elsewhere in the genome (BLASTP analysis). A total of 11 enzymes for lipid A biosynthesis encoded by *htrB*, *kdsA*, *kdsB2*, *kdtA*, *lnt*, *lpxA*, *lpxB1*, *lpxC*, *lpxD1*, *lpxD2* and *lpxK* were present across the Thai Paidjan and Dadas genomes, but both lacked *lpxB2* and *kdsB1* (BLASTP analysis) that are found in *L. interrogans* serovar Lai strain 56601 [6].

### Average nucleotide identity (ANI) and phylogenomic analysis

The results from the JSpecies analysis showed that the ANI percentages among the genus *Leptospira* varied widely from 64.17 to 99.19% for pairwise genome comparisons in the pathogenic, intermediate, and non-pathogenic *Leptospira* groups. For example, genomic comparison between *L. wofflii* and *L. biflexa* revealed 64.17% nucleotide similarity, whereas *L. interrogans* serovar Copenhageni and *L. interrogans* serovar Djasiman had 99.19% nucleotide similarity (Additional file 3: Table S1). Moreover, in 13 representative *L. interrogans* strains, the ANI values gave a similarity percentage of more than 98%. Strains CUDO5 and CUDO8 had the highest ANI values to each other (99.08%).

**Fig. 5:**
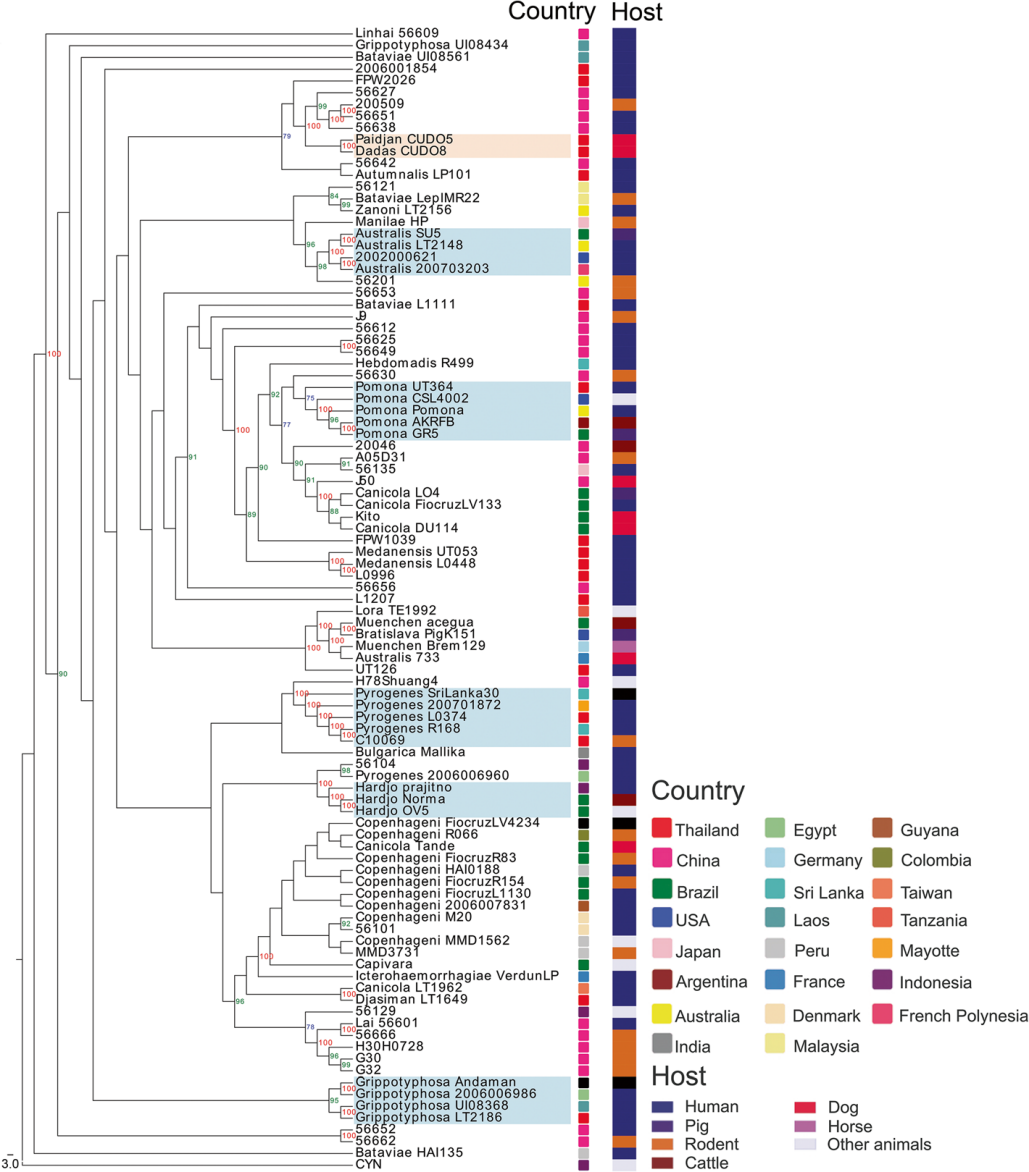
Whole genome core-single nucleotide polymorphisms (core-SNPs) phylogeny of *L. interrogans* based on the maximum likelihood method with 1000 bootstrap replicates. The core-SNPs phylogenomic analyses was visualized by EvolView [63]. The tree shows that *L. interrogans* serovar Paidjan strain CUDO5 and serovar Dadas strain CUDO8 were placed in the same branch with a bootstrap percentage of 100 as highlight in peach color. Serovar specific clades with bootstrap percentage of more than of 75 are highlight in blue color, including serovar Australis, Grippotyphosa, Hardjo, Pomona and Pyrogenes. *L. kirscheneri* serovar Cynopteri strain 3522 CT (CYN) was used as an outgroup in this study

For phylogenomic analysis of *L. interrogans* strains, a total of 87,442 concatenated core-SNPs from the 97 genomes (Additional file 3: Table S2) were used to generate a maximum likelihood phylogenetic tree with the GTR nucleotide substitution model. All strains were obtained from different geographical regions and hosts. The phylogenetic tree revealed two defining features: serovar specific branches, and a connectivity of human and animal isolates. Some serovars were clustered in the same clades, with bootstrap percentage of more than 75 supported, including serovar Australis (strains 200,703,203, SU5 and LT2148), serovar Grippotyphosa (strains Andaman, 2006006986, UI08368 and LT2186), serovar Hardjo (strains Prajitno, OV5 and Norma), serovar Pomona (strains UT364, GR5, AKRFB, Pomona and CSL 4002), and serovar Pyrogenes (strains 200701872, R168, L0374 and Sri Lanka 30). These strains came from different geographical locations. Notably, an intermingling of human and animal isolates was identified, particularly in the cluster of serovar Australis (human and pig), Hardjo (human and cattle), Pomona (human, pig and cattle) and Pyrogenes (human and rodent). *L. interrogans* serovar Paidjan strain CUDO5 and serovar Dadas strain CUDO8 were grouped in the same branch, with a bootstrap percentage of 100. Moreover, strains CUDO5 (serovar Paidjan) and CUDO8 (serovar Dadas) isolated from the urine of asymptomatic dogs were clustered on the evolutionary tree with leptospiral isolates recovered from humans in Thailand and China (Fig. 5).

### Pan-genome and core-genome analysis, and identification of strain-specific genes in the two Thai canine strains

**Fig. 6:**
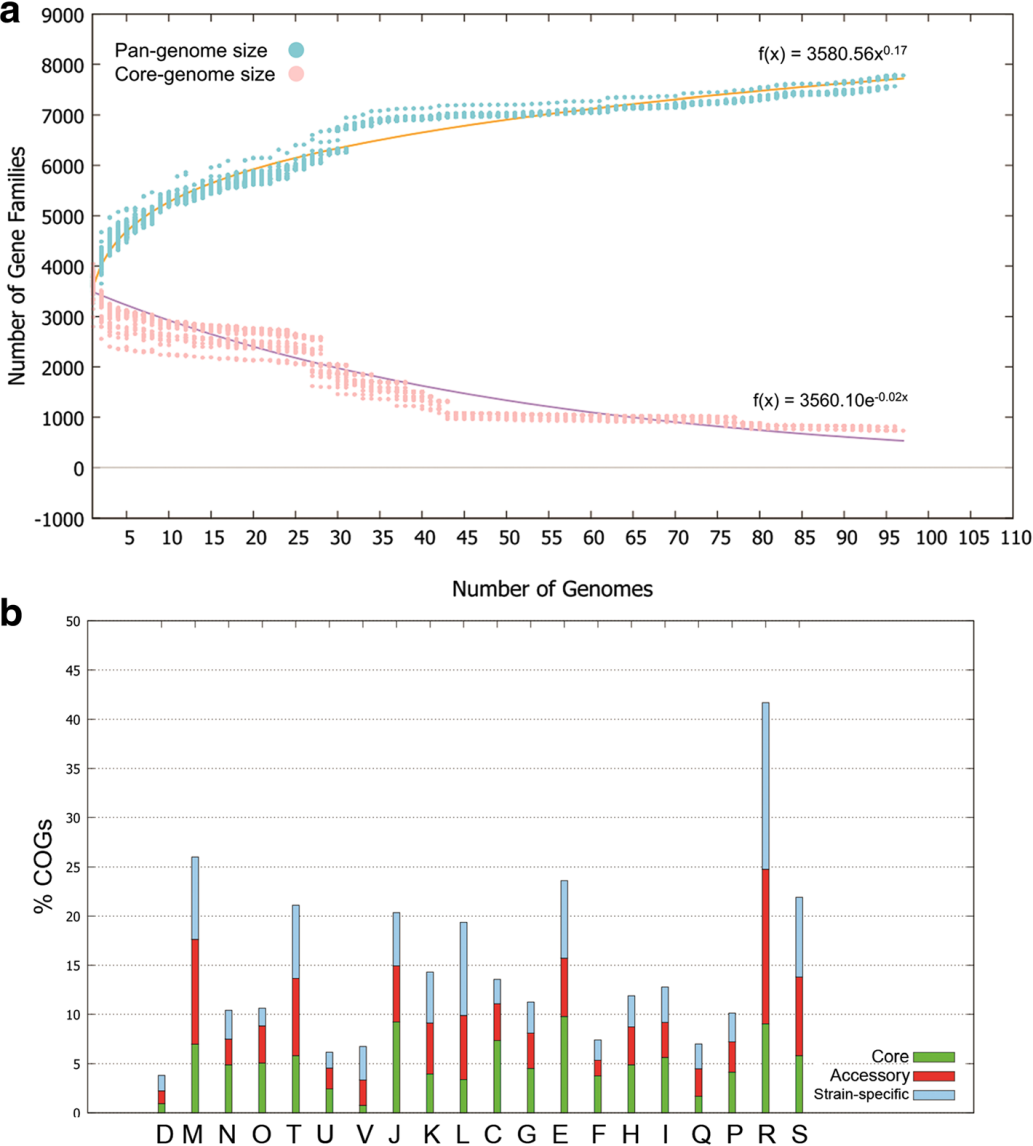
Pan-genome analysis of *L. interrogans* (*n* = 97) using the BPGA pipeline. The power and exponential law equations were used to create pan- and core-genome curves of *L. interrogans* (panel **a**). The COG categories distribution of core, accessory and strain-specific genes in 97 *L. interrogans* strains are shown in panel **b**, and according to Heap’s law the *Leptospira* genome was predicted to be open. The COG categories are denoted as follows: D (Cell cycle control, cell division, chromosome partitioning), M (Cell wall/membrane/envelope biogenesis), N (Cell motility), O (Posttranslational modification, protein turnover, chaperones), T (Signal transduction mechanisms), U (Intracellular trafficking, secretion, and vesicular transport), V (Defense mechanisms), J (Translation, ribosomal structure and biogenesis), K (Transcription), L (Replication, recombination and repair), C (Energy production and conversion), G (Carbohydrate transport and metabolism), E (Amino acid transport and metabolism), F (Nucleotide transport and metabolism), H (Coenzyme transport and metabolism), I (Lipid transport and metabolism), Q (Secondary metabolites biosynthesis, transport and catabolism), P (Inorganic ion transport and metabolism), R (General function prediction only), and S (Function unknown)

Using the power fit law equation, it was calculated that b = 0.167923, and the expected size of the *L. interrogans* pan-genome was 7782 gene families. The pan-genomes tended to increase after each new genome addition (Fig. 6a), and were assumed to be open in this analysis (*n* = 97). In contrast, the core-genome gradually decreased and became constant after 80 genomes were analyzed (Fig. 6a).

The core gene set across the *L. interrogans* strains (*n* = 97) was made up of 734 gene families, whilst the accessory gene set varied from 2062 to 3303 gene families (Additional file 3: Table S3). The functional classification of the gene repertoire in core- and strain-specific genes according to COG categories was different. Core-genes encoding for proteins related to category J (Translation, ribosomal structure and biosynthesis), E (Amino acid transport and metabolism) and R (General prediction only) were abundant among the total core-genes identified, whereas strain-specific genes encoding for proteins involved in categories R (General function prediction only), L (Replication, recombination and repair) and M (Cell wall/membrane/envelope biogenesis) were over-assigned among this set of genes. The COG distribution of the core-, accessory and strain-specific genes are shown in Fig. 6b.

The pan-genome analysis of the 97 *L interrogans* strains identified genes that were only found in the two Thai canine strains (i.e. were specific to them). *L. interrogans* strain CUDO5 contained eight strain-specific genes, of which seven could not be classified by eggNOG; hypothetical protein. One gene was found using an NCBI database search, and its predicted product resembled a type III restriction endonuclease. In strain CUDO8, BPGA identified three strain-specific genes, of which two were classified as hypothetical proteins, and the other was assigned as phage terminase large subunit.

### Positive selection in the *L. interrogans* genome and mapping on protein tertiary structure

**Fig. 7:**
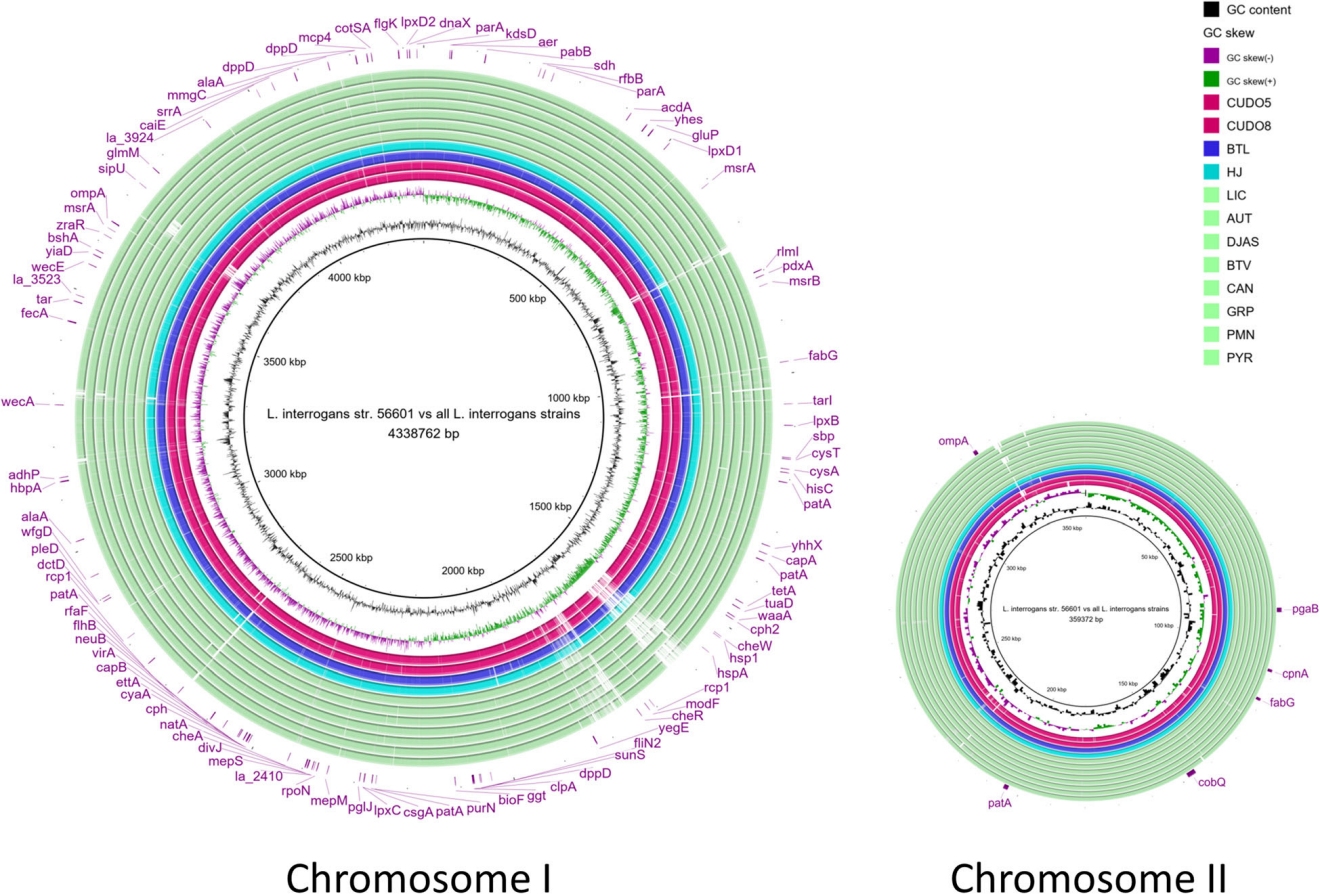
Circular genome map of *L. interrogans* serovar Lai strain 56601 and the other 13 representative strains studied, with the location of 101 predicted virulence genes under positive selection marked. Comparative sequence analysis was performed with BRIG [76] using *L. interrogans* strain 56601 as a reference (thick black innermost ring) against other representative related *L. interrogans* strains. The second inner ring indicates GC contents (black color) followed by the GC skew (purple and green colors). Each colored ring shows the host origin in each strain of chromosomal DNA of *Leptospira* presented as follows: magenta for dogs, blue for pigs, cyan for cattle and light-green for humans. The outermost labels show the location of 101 predicted virulence genes under positive selection in *L. interrogans*. The intensity of the ring color represents the percentage of nucleotide identity from 50 to 90

According to GET_HOMOLOGUES, a total of 2721 single-copy orthologous genes were shared among the 13 representative *L. interrogans* strains, and these were defined as core genes. After eliminating incomplete sequences due to frameshift mutations, insertions and deletions, 2408 protein-coding sequences were left. Of these, 56.36% of the encoded proteins had known function and the rest were hypothetical proteins. This study focused only on proteins of known function. Based on the LRT statistic for the site-model, 352 genes were under significant positive selection (FDR < 5%). Genes under positive selection were further identified based on functional classification and on whether they encoded known virulence factors, using the COG and VFDB categories respectively. In relation to functional classification, COG classified protein-coding genes involved with category M (cell wall/envelope biosynthesis), E (amino acid transport), H (coenzyme transport and metabolism), T (signal transduction mechanism) and J (Translation, ribosomal structure and biogenesis) were significantly over-represented among the genes showing evidence of positive selection (*P* < 0.05, one-side binomial test) (Additional file 4: Table S1). Of these genes under positive selection, 101 were predicted as encoding putative virulence factors. Most of the genes in the three groups adhesion, anti-phagocytosis and iron uptake system were significantly over-represented (*P* < 0.05, one-side binomial test) (Additional file 4: Table S2). Moreover, another 74 genes showed evidence for positive selection and recombination (Additional file 4: Tables S3 and S4, Additional file 5: Figure S1). Interestingly, *colA* encoding for collagenase was identified amongst these, and this had an amino acid sequence homology of 29.19% to the enzyme in *Clostridium perfringens*. Positional mapping of 101 predicted virulence genes under positive selection is shown in Fig. 7, and a summary of statistical analyses relating to positive selection for 18 representative virulence genes related to adhesion, anti-phagocytosis, iron uptake system, LPS biosynthesis, exotoxin and motility of *L. interrogans* is presented in Table 2.

**Table 2:** Statistical summary of 18 virulence-associated genes under positive selection in 13 representative *L. interorgan* strains

Gene	COG categories^a^	Gene annotation	ω^b^	2Δℓ^c^	*q*-value
Adhesion					
*kdsD*	M	Arabinose 5-phosphate isomerase	15.32026	10.347320	0.011652800
*ompA*	M	Outer membrane protein A	17.12571	20.820944	0.000130800
*yiaD*	M	Outer membrane protein A	18.64214	23.296514	0.000046900
*rfaF*	M	Glycosyl transferase, family 9	20.83838	13.943466	0.002602300
*la_2410*	M	Glycerol-3-phosphate cytidylyltransferase	20.63787	11.376946	0.007541200
*sunS*	M	Glycosyl transferase, family 2	77.84603	23.733642	0.000039100
*patA*	M	Membrane bound *O*-acyltransferase (MBOAT) family protein	33.76757	34.295972	0.000000426
Anti-phagocytosis					
*rmd*	M	NAD-dependent epimerase dehydratase	15.67082	13.845838	0.002710400
*capB*	M	Capsule biosynthesis protein	10.13343	16.584998	0.000831400
*capA*	M	Capsule biosynthesis protein	26.72717	7.6514580	0.037726700
Iron uptake system					
*fecA*	M	TonB-dependent receptor plug domain	14.34022	31.619632	0.000001330
*hbpA*	P	TonB-dependent receptor	8.341820	10.766942	0.009678900
*modF*	P	ABC transporter	10.62873	8.1123800	0.030958000
LPS biosynthesis					
*lpxD1*	M	UDP-3-*O*-(3-hydroxymyristoryl) glucosamine *N*-acyltransferase	65.82909	9.594336	0.016131033
Exotoxin					
*tlyA*^d^	J	Hemolysin A	61.51328	30.989188	0.000001670
Motility					
*fliN2*	N	Flagella motor switch protein	31.01218	35.57534	0.000000249
*flgK*	N	Flagella hook-associated protein	16.72326	15.544766	0.001312461
*flhB*	N	Polar flagella protein	999.0000^e^	22.055182	0.000076800

^a^The abbreviations for COG categories are defined as: M (Cell wall/membrane/envelope biogenesis), P (Inorganic ion transport and metabolism), J (Translation, ribosomal structure and biogenesis) and N (Cell motility)

^b^ω is the ratio of *d*N/*d*S under positive selection (model M2a)

^C^2Δℓ is likelihood ratio test (LRT) statistic

^d^*tlyA* is not found in VFDB database but this gene has been shown to be a virulence factor in pathogenic *Leptospira* [24]

^e^All substitutions of *flhB* are non-synonymous and *d*S = 0; therefore the ω value is undefined and represented as 999 in the table. According to CODEML developer, LRT is not affected by *d*S in this analysis as shown by a significant *q*-value result

To obtain more insight into positive selection based on protein tertiary structure, amino acid residues under positive selection were mapped on 3D-modeled protein structures. Of the 101 genes under positive selection and predicted to be related to putative virulence in *Leptospira*, four representative proteins that have been experimentally confirmed to be virulence factor of pathogenic *Leptospira* were selected for this analysis: hemolysin A (encoded by *tlyA*); TonB-hemin binding receptor (encoded by *hbpA*); flagella motor switch protein (encoded by *fliN2*); and UDP-3-*O*-(3-hydroxymyristoryl) glucosamine *N*-acyltransferase (encoded by *lpxD1*). These all had values of ω > 1, significant *q*-values, and a high confidence of obtaining 3D-protein-based homology predictions. The *tlyA*, *hbpA*, *fliN2* and *lpxD1* genes encoded proteins made up of 259, 776, 178 and 340 amino acid residues, respectively.

**Fig. 8:**
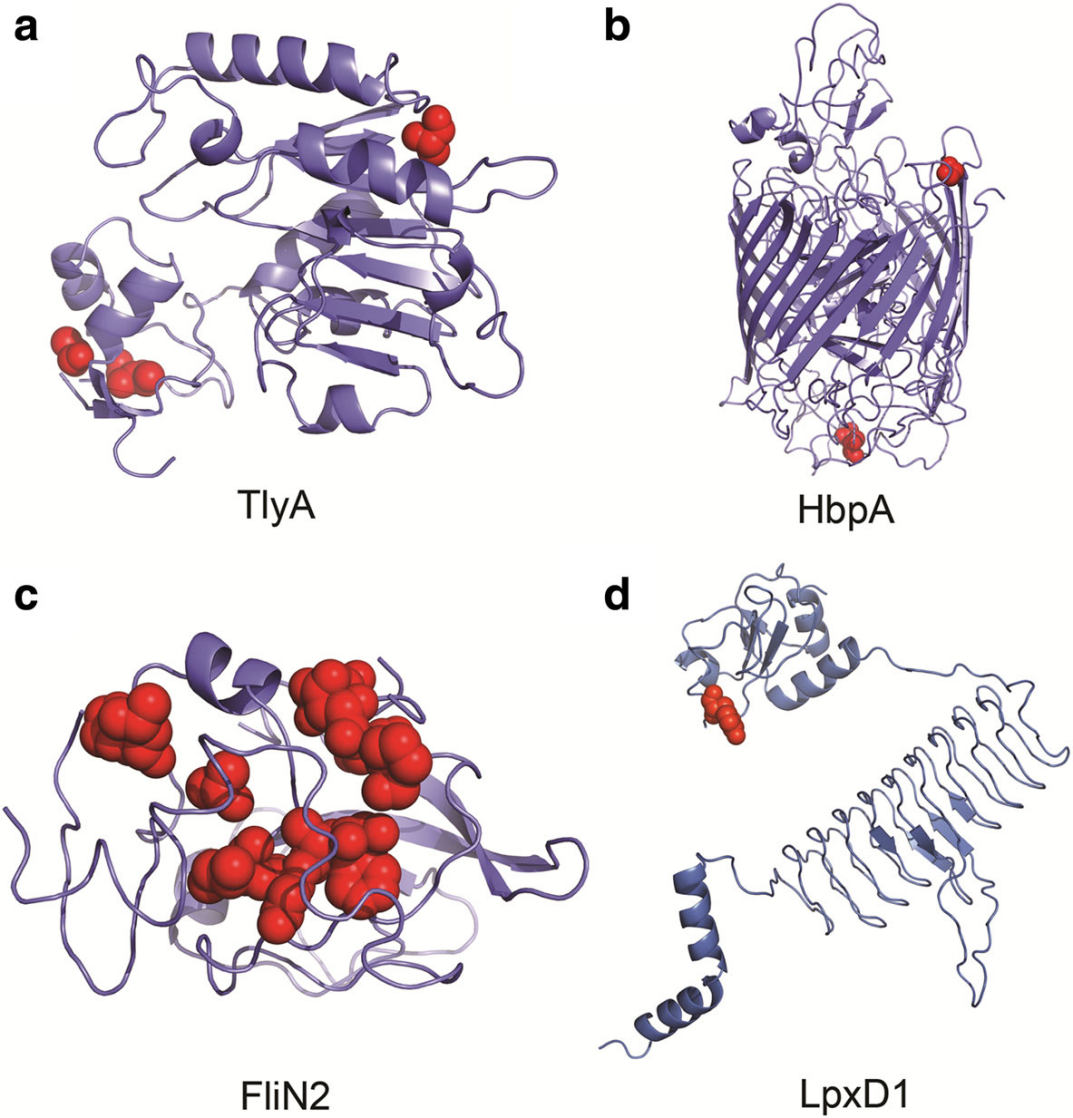
Three-dimensional protein models of Hemolysin A (encoded by *tlyA*: panel **a**), Ton-B hemin binding receptor (encoded by *hbpA*: panel **b),** Flagella motor switch protein (encoded by *fliN2*: panel **c**), and UDP-3-*O*-(3-hydroxymyristoryl) glucosamine *N*-acyltransferase (encoded by *lpxD1*: panel **d**) under positive selection in *L. interrogans*. Sites of strong positive selection are highlighted as red spheres (posterior probability > 0.95)

The results from the BEB analysis revealed that three amino acids in *L. interrogans* TlyA (amino acid positions 40, 43 and 258), two in HbpA (positions 240 and 395), 11 in FliN2 (positions 54, 58, 63, 65, 67, 68, 69, 70, 72, 74 and 75) and one in LpxD1 (position 47) were subjected to positive selection with a high posterior probability (PP > 0.95). According to the protein tertiary mapping by PyMOL, positive selection of TlyA occurred in the loop connecting two α-helices and the end of the C-terminus (Fig. 8a), whereas for HbpA, positive selection was present in the loop connecting two β-pleated sheets (Fig. 8b). In FliN2, positive selection occurred in the loop linking two α-helices (Fig. 8c), while for LpxD1 positive selection was detected within a β-plated strand (Fig. 8d). Moreover, at more than 90% confidence, Phyre2 modeled *L. interrogans* TlyA as sharing 37% amino acid identity with a putative hemolysin of *Streptococcus thermophiles*; FliN2 had 44% amino acid identity to a flagella motor switch protein homolog in *Salmonella* Typhimurium; HbpA shared 14% amino acid identity with a ferripyoverdine receptor in *Pseudomonas aeruginosa*; and LpxD1 shared 26% amino acid identity with UDP-3-*O*-(3-hydroxymyristoryl) glucosamine *N*-acyltransferase in *Pseudomonas aeruginosa*. The Phyre2 investigator tool showed that none of the amino acid residues under positive selection in TlyA, HbpA, FliN2 or LpxD1 occurred in the pocket sites.

## Discussion

The present study reports the first genomic characterization of *L. interrogans* strains isolated from dogs in Thailand, and presents a comparative genome analysis of these with the published genomes of another 12 *Leptospira* species. Moreover, a genome-wide scan of genes under positive selection, focusing on potential virulence factors of *L. interrogans*, was undertaken to obtain insights into genetic diversity, molecular pathogenesis and molecular adaptation of the *Leptospira* strains.

*L. interrogans* serovar Paidjan strain CUDO5 and *L. interrogans* serovar Dadas strain CUDO8 were isolated from the urine of asymptomatic dogs in an area of Thailand with a high frequency of human leptospirosis, and a 9.92% prevalence in animals. Evidence from previous *rrs* phylogenetic analysis has revealed close genetic links between isolates from animals and humans in this area [19]. Therefore, theses strains have epidemiological importance and potentially may pose a risk to public health in the region. The availability of genome sequences from both Thai *Leptospira* serovars provides useful new information relating to disease diagnosis and prevention.

Analysis of the genomes of the two Thai strains demonstrated that they had similar numbers of CDSs and sub-system categories to other analyzed *L. interrogans* strains. Most of the genes encoded proteins responsible for basic biological processes such as transcription, translation, cell wall synthesis and motility, and the findings confirm the high conservation of CDSs among *L. interrogans* strains.

Phages are viruses that infect specific bacterial hosts, and are abundant in the environment [20]. Prophage-related genes were found in the *Leptospira* genomes, presenting as incomplete phages without any obvious association with pathogenicity islands or antimicrobial resistant genes, but with diverse prophage types (Additional file 1: Table S3). In this study, *Sphingomonas* phage (PHAGE_Sphing_PAU_NC_019521) was found in 11 of the 13 representative *L. interrogans* strains, and this phage potentially could be exchanged between *L. interrogans* and *Sphingonomas paucimobilis* in shared environments such as in water sources [21].

The CRISPR-Cas system is defined as part of the bacterial adaptive immune system. This system can remember and destroy phages and plasmids invading the host cell by producing specific crRNA against these elements [18]. Our results are in agreement with a previous study where the CRISPR-Cas system was found only in intermediate and pathogenic group strains, which harbored at least one subtype of CRISPR1, whereas non-pathogenic *Leptospira* species did not have this system [6]. The CRISPR-Cas system has been thought to be a necessary functional system in pathogenic *Leptospira* [6]. The strains from dogs in this study possessed two CRISPR1 loci (subtypes I-B and I-C). CRISPR1 subtype I-B targets foreign DNA invasion, while the function of subtype I-C is still uncharacterized. These two loci of the CRISPR-Cas system also are found in *Pyrococcus furiosus*, suggesting its ability to use crRNA to defend against a wide range of foreign genetic material, including both DNA and RNA [22]. Various crRNAs targeting either plasmids or phages in various species were identified in *L. interrogans* serovar Dadas strain CUDO8 (*n* = 40) and to a lesser extent in *L. interrogans* serovar Paidjan strain CUDO5 (*n* = 31) (Additional file 1: Table S4). The difference suggests that CUDO8 may have been subjected to infection with more phages and plasmids than CUDO5. The fact that the plasmid of *L. interrogans* serovar Linhai strain 56609 was found in CUDO5 and CUDO8 might reflect their shared evolutionary history, or transfer of the plasmid during their occupation of shared microenvironmental niches before their dissemination to different hosts. On the other hand, it should be noted that crRNAs targets predicted by bioinformatics programs quite commonly have non-specific or coincidental hits (both plasmid and phage), especially when querying with short sequences. Therefore, a CRISPR interference assay is required to validate the crRNAs target results relating to interference with foreign genetic material in *Leptospira* [23].

The presence of virulence genes associated with the pathogenesis of leptospirosis have been used to divide *Leptospira* species and strains into three groups: pathogenic, intermediate and non-pathogenic [24]. In this study, we identified virulence factors present in strains CUDO5 and CUDO8 genomes using the VFDB database. However, VFDB searching has some limitations since virulence genes/proteins with confirmed roles in other bacteria might not reflect their function in strains CUDO5 and CUDO8. Therefore, a combination search with orthologous virulence genes with established roles in acute and chronic leptospirosis also was undertaken. All pathogenic *Leptospira* strains contained genes encoding catalase, collagenase, TonB-hemin binding receptor, immunoglobulin-like protein B and sphingomyelinases, which all are confirmed as virulence markers during the early stage of infection [24]. On the other hand, intermediate and non-pathogenic *Leptospira* lack many of these virulence genes: their acquisition may reflect an evolutionary process favoring host infection and disease [6], whilst loss of these genes may result in attenuation and reduction in virulence. Although strains CUDO5 and CUDO8 have genes with confirmed roles as virulence factors, they were isolated from the urine of asymptomatic dogs. Their ability to establish persistent infections with long-term kidney colonization is likely to reflect a balance of gene content and expression that favors an advantageous carrier state - maximizing the opportunity for further transmission via urine.

Comparative genome analysis can be used to understand genomic diversity and provide insights into biological functions and evolution [25]. Using the power law exponents of Heap’s law, our data indicated that *L. interrogans* strains have an open pan-genome [6, 7]. This finding suggests that the gene pool of the spirochetes can expand, and the observed genomic plasticity probably results from multiple ways of incorporating new genetic material into their genomes such as through horizontal gene transfer and/or replication of advantageous genes via gene duplication [7]. This phenomenon is beneficial in relation to adaptations resulting in increased infectivity and survival in a broad range of mammalian hosts and the environment [1].

The *Leptospira* species that were analyzed were separated into three groups using DNA-DNA hybridization as well as by virulence gene profiles [6]. The 13 representative strains of *L. interrogans* had ANI values of over 98%, suggesting that nucleotide sequences of *L. interrogans* strains are mostly conserved, even though the strains were of different serovars and were from different origins and epidemiological units. The ML-based core-SNPs phylogenetic analysis identified both distinct and clustered *Leptospira* serovars in the same branch of the phylogenetic tree. This finding reflected the diversity of the *Leptospira* strains, even amongst those coming from the same geographical origins. It also inferred different accumulations of SNPs in the core genome in each strain. Moreover, there were instances of connectivity between human and animal *Leptospira* isolates, providing genetic evidence supporting the occurrence of disease maintenance in animals and cross-species transmission. Notably, the phylogenetic tree confirmed that the two Thai canine strains were closely related to each other. However, the serotyping results revealed that they belonged to different serogroups and serovars (serogroup Bataviae serovar Paidjan for strain CUDO5 and serogroup Grippotyphosa serovar Dadas for strain CUDO8). Consistent with the *rfb* locus analysis, serovars Paidjan and Dadas had gene variations in these regions. Nevertheless, genes association with rhamnose biosynthesis (*rfb ABCD*) and rhamnose containing glycan (*rfbB* and *galE*) were distributed in both serovars. Previously, the LPS of pathogenic *Leptospira* has been shown to contain more rhamnose than intermediate *Leptospira*, and moreover specific sugars were found related to particular serovars [26]. This finding was in agreement with our results in the case of serovar Dadas that contained three additional genes associated with capsular heptose biosynthesis. Furthermore, the gene encoding aminotransferase PglE that was found in both serovars might play a crucial role in creating novel sugar composition during the glycosylation process of outer membrane proteins [27]. The three aminotransferases belonging to the DegT family in serovar Paidjan might be associated with LPS O-antigen side chain biosynthesis, leading to differences between the serological characteristics of the serovars [28]. These findings provided possible explanations for the difference in LPS composition and outer membrane protein post-translational modifications between serovars Paidjan and Dadas. However, more experiments are required to compare the relationship of the genes in the *rfb* locus, sugar composition, sugar ramification and structural determination of the LPS, since there are large variations and complexities in this material [1]. A previous investigation of the LPS of pathogenic and intermediately pathogenic *Leptospira* species using multiple methods including LPS extraction, gas chromatography-mass spectrometry (GC-MS), and electron microscopy revealed significant differences in terms of carbohydrate and fatty acid composition, including in *Leptospira* isolated from common reservoir hosts [26]. The presence of large quantities of a specific sugar in a particular serovar is believed to play an important role for survival and colonization in certain environments [26]. Therefore, a combination of genomic detail and biochemical and structural analysis will allow a better understanding of LPS biology in the adaptation strategies in these two pathogenic *Leptospira*.

In bacteria, strain-specific genes are associated with specific functions and niche adaptations [25]. Strains CUDO5 and CUDO8 harbored strain-specific genes compared to the other strains in this study (*n* = 97), but these were mostly predicted as hypothetical proteins with unknown functions. Nevertheless, strain-specific genes of known function were found in these strains, consisting of; a type III restriction endonuclease and phage terminase large subunit. The function of these genes may be related to the pathogenesis of *Leptospira*. Notably, type III restriction endonucleases are functionally associated with defense mechanisms against invading foreign DNA. Therefore, this gene possibly functions to protect *L. interrogans* from phage infection, and could modulate the genetic exchange barrier [29]. As no complete phage was reported in this study, the cryptic prophage including phage terminase large subunit might indicate the existence of a prior phage infection that was probably degraded by the CRISPR-Cas system and/or a restriction modification system, and was subsequently trapped in the *Leptospira* chromosome [30]. Although the role of phage terminase large subunit in the *Leptospira* strains was not established, cryptic prophages in *E. coli* contribute to survival in adverse environment conditions [31]. Therefore, this cryptic prophage might help *Leptospira* to cope with stress in new hosts and environments.

Beneficial mutations in bacterial genes under positive selection can assist in molding bacterial-host adaptation [11], whilst recombination helps positive selection to become fixed in the population [32]. Our investigation of positive selection among the genomes of 13 representative *L. interrogans* strains focused on genes encoding virulence-associated proteins. Genes under significant positive selection included 101 predicted virulence-associated genes and 352 genes with various functions, including those involved in cell wall/envelope biosynthesis; amino acid transport and coenzyme transport and metabolism; and signal transduction and cell translation: these function in binding and transport of essential nutrients in the early stages of infection and bacterial replication. Using the VFDB database, the genes under positive selection were grouped by virulence functions consisting of adhesion, anti-phagocytosis and iron uptake systems. In *Leptospira* infection an initial step is adhesion at the site of abrasion or on mucous membranes. In general, several adhesion factors are responsible for attachment to a variety of cell surfaces, such as the extracellular matrix (ECM), complement factors, macrophages, fibroblasts, endothelium cells and kidney cells [6, 33]. Therefore, positive selection could enhance adhesion ability and assist *Leptospira* in spreading throughout the body during the initial stages of infection. Moreover, positive selection was found on binding sites involved in immune recognition. Protein conformational changes may be part of a strategy for anti-phagocytosis by using surface adhesion-related proteins to trap complement factors and in turn avoid opsonization and IgG deposition on the leptospiral surface membrane [34]. In this study, four genes encoding experimentally confirmed virulence factors in pathogenic *Leptospira* were selected to construct 3D-structure models with positive selection site mapping to investigate possible functional implications of changes to the proteins (genes *tlyA*, *hbpA*, *fliN2* and *lpxD1*). Specific amino acids in each molecule were identified as being under positive selection, but the significance of these in relation to their structure and function was not investigated further. Genes related to iron uptake systems were under positive selection, including the *tlyA* gene encoding hemolysin A and *hbpA* encoding the TonB-hemin binding receptor for which the structures were analyzed. These genes may provide a synergistic effect for iron acquisition from red blood cells during *Leptospira* infection [35]. Previously, endoflagella and protein-related LPS biosynthesis were confirmed as true virulence factors in pathogenic *Leptospira* [36]. In the present study, we found positive selection on genes associated with endoflagella, including *fliN2* encoding for flagella motor switch protein, *flgK* encoding for flagella hook-associated protein, and *flhB* encoding for polar flagella protein. This positive selection is likely to be important for motility of *Leptospira,* allowing translocation, penetration and dissemination in host tissues [37, 38]. The gene *lpxD1* encoding for UDP-3-*O*-(3-hydroxymyristoryl) glucosamine *N*-acyltransferase that is involved with lipid A biosynthesis as part of the LPS structure also was subjected to positive selection. Studies have shown that alterations to the gene *lpxD* contribute to modification in lipid A, and subsequently influence outer membrane integrity when bacteria are subjected to changing temperatures [36, 39]. Therefore, the positive selection on *lpxD1* may be involved in assisting *Leptospira* to adapt to temperature differences in mammalian hosts. Additionally, mutations in *lpxD1* have been considered to be involved with kidney and liver colonization by *Leptospira* in an asymptomatic animal model. It also has been suggested that pathogenic *Leptospira* have lipid A modification strategies to evade immune response in the early stage of infection, leading to development of chronic leptospirosis in mammalian hosts [40]. The gene *colA* encoding for collagenase (ColA) showed evidence of both positive selection and recombination in this study. ColA has been experimentally confirmed as a virulence factor in *Leptospira* [41]. Positive selection in *colA* might promote *Leptospira* invasiveness in the host body, whilst recombination of this gene might facilitate spread of advantageous mutations to other *Leptospira* [32, 41].

Pathogenic *Leptospira* have the ability to cause chronic infection by evading the host immune response [42]. Several different strategies have been described for immune evasion including biofilm formation, an increase in LPS O-antigen, and down-regulation of outer membrane proteins [43–45]. Moreover, during prolonged infections immune pressures acting on such pathogens are likely to select for advantageous genetic variation within the genome, leading to accumulation of mutations that increase survival fitness or that cause loss of function in virulence factors [46]. The balance of these genetic alterations may contribute to the strain’s adaptation and establishment of a reservoir/carrier state in an animal. Our study has improved understanding of genetic adaptation in this important zoonotic pathogen and provided promising gene targets for further research. This should lead to development of better control strategies, such as improved vaccines and more accurate diagnostic tests for use in the region.

## Conclusions

This study provided genomic data and characterization of Thai *L. interrogans* serovar Paidjan stain CUDO5 and *L. interrogans* serovar Dadas strain CUDO8 isolated from the urine of asymptomatic dogs; furthermore, comparative genomic analysis with the genomes of 97 *L. interrogans* strains were undertaken and revealed an open pan-genome for the strains. Genes with established roles in acute and chronic leptospirosis were found in both Thai canine strains. Core-SNPs phylogeny confirmed their close genetic relatedness, but the *rfb* locus analysis identified genetic variations between them that were consistent with the different serotyping results. Genes under positive selection were identified, including genes encoding factors involved in motility, temperature adaptation, and iron acquisition: these may be involved in bacterial dissemination, thermal adaptation and nutrient uptake. Further investigations into the functions and expression of these genes, including in vivo experiments are required to expand these findings.

## Methods

### Bacterial strains, growth conditions and DNA extraction

With approval by the Chulalongkorn University Animal Care and Use Committee (CU-ACUC; Protocol number 1531075) and the Institutional Biosafety Committee (CU-VET-IBC; Protocol number 18310430), two Thai *L. interrogans* strains named CUDO5 and CUDO8 that were isolated from the urine of asymptomatic dogs in Nan province, Thailand (year 2014), were used in this study. The bacteria were identified to species level by partial *rrs* sequencing and comparative phylogeny, and were assigned as novel sequence types (ST) ST26 and ST33 respectively by multilocus sequence analysis of seven housekeeping genes (*caiB*, *glumU*, *mreA*, *pfkB*, *pntA*, *sucA* and *tpiA*) [19].

*L. interrogans* strains CUDO5 and CUDO8 that were passaged less than five times were kept as stock culture and stored at − 80 °C before use. A 500 μL volume of each thawed stock was cultured in 5 mL of liquid Ellinghuasen-McCullough-Johnson-Harris (EMJH) medium with 3% rabbit serum, and incubated at 30 °C for two weeks. Subsequently, 300 μL of the cultures were spread on blood agar and incubated at 37 °C for two days to verify lack of contamination with other bacteria by visual inspection of surface growth. To purify and collect the leptospires, the suspensions were centrifuged at 15,000×*g* at 4 °C for 15 min and then the genomic DNA was extracted from the cell-pellet using the Wizard® Genomic DNA Purification Kit (Promega, USA) following the manufacturer’s instructions. The quality and quantity of the DNA were measured using a spectrophotometer to obtain the A260/230 ratio (1.51 for CUDO5 and 1.75 for CUDO8) and the A260/280 ratio (1.80 for CUDO5 and 1.83 for CUDO8) (NanoDrop™, Thermo Fisher Scientific, USA) and Qubit™ Fluorometric Quantitation (Thermo Fisher Scientific, USA), respectively. Genomic DNA was stored at − 20 °C before library preparation and genome sequencing.

### Serotyping

Serotyping of the *L. interrogans* strain CUDO5 and CUDO8 was performed at the OIE National Collaborating Centre for Reference and Research on Leptospirosis, The Netherlands. A total of 43 polyclonal rabbit antisera (pAbs) raised against strains of all pathogenic and non-pathogenic *Leptospira* serogroups were used in the microscopic agglutination test (MAT) to determine the serogroup (Additional 5, Table 1). Subsequently, the isolates were further identified to the serovar level using monoclonal antibodies (mAbs). Strains CUDO5 and CUDO8 were subjected to serovar typing employing the whole set of mAbs derived from *Leptospira* serogroup Bataviae serovar Losbanos strain LT 101–69: F129C2, F129C3, F129C4, F129C6, F129C7, F129C9, F129C15, F129C18, F129C19, F129C20, F129C24, F129C25 and F129C26, and *Leptospira* serogroup Grippotyphosa serovar Grippotyphosa strain Moskva V, serovar Vanderhoedeni strain Kipod 179 and serovar Grippotyphosa strain Dutch M: F71C2, F71C3, F71C9, F71C13, F71C16, F71C17, F164C1, F165C1, F165C2, F165C3, F165C7, F165C8 and F165C12, respectively (https://leptospira.amc.nl/leptospirosis-reference-centre/typing-with-monoclonal-antibodies/) (Additional file 6: Tables S1 and S2).

### Genome sequencing, de novo assembly and annotation

DNA from *L. interrogans* strains CUDO5 and CUDO8 was subjected to whole genome sequencing and de novo assembly, as previously described [47]. Briefly, DNA libraries were prepared from genomic DNA using the Nextera XT DNA Library Preparation kit (Illumina, USA), following the manufacturer’s protocol (average insert size = 375 bp). Genome sequencing was carried out using Illumina MiSeq with a 250 paired-end run cycles. As a consequence of the substantial genetic variation in the serovar determinant region, and to avoid bias using reference mapping, de novo genome assembly was conducted using the A5-Miseq pipeline that consisted of 5 steps including read cleaning and base error correction, contig assembly, crude scaffolding, mis-assembly correction and final scaffolding [48]. Scaffolds were oriented and ordered against the whole genome sequence of *L. interrogans* serovar Lai strain 56601 using ABACAS [49]. Gaps among the scaffolds were closed using the IMAGE program [50]. The draft genomes of *L. interrogans* strains CUDO5 and CUDO8 were annotated using rapid prokaryotic genome annotation (PROKKA) [51] and Rapid Annotation using Subsystem Technology (RAST) version 4.0 [52]. Prophage sequences were identified using the PHASTER web server with the default setting [53]. Clustered regularly interspaced short palindromic repeats (CRISPRs) and Cas regions were examined by CRISPRone [54]. The spacer results from CRISPRone were further analyzed for CRISPR RNA (crRNA) targets by searching against the GenBank-Phage and RefSeq-Plasmid databases using CRISPRTarget [55]. To predict overall putative virulence genes, the genomes of strains CUDO5 and CUDO8 were search against the virulence factor database (VFDB) [56]. Moreover, a list of previously confirmed virulence genes involved with acute and chronic *Leptospira* infections was generated based on a literature review of in vitro and in vivo experimental models [24, 36, 57] (Additional file 1: Table S7). From these, 33 representative genes with good data support were selected for gene orthologous identification using amino acid sequences retrieved from UniProt database: these sequences then were subjected to a BLAST search against all proteomic sequences of representative *Leptospira* strains using BLASTP analysis with an e-value threshold of 1e-06 and protein identity of more than 40% [58]. A heat map of the presence and absence of virulence genes among *Leptospira* strains was constructed using Clustergrammer with the default parameter [59].

Draft genome sequences of *L. interrogans* strains CUDO5 and CUDO8 were submitted to NCBI’s database as Whole Genome Shotgun (WGS) sequences under the accession numbers NKYH02000001.1 and NKYG00000001.1, respectively [47].

### Average nucleotide identity (ANI) and phylogenomic analysis

To define genome relatedness and similarities among members of the genus *Leptospira*, 22 representative genomes derived from 19 pathogens, two intermediate and one non-pathogen were recruited to be analyzed together with the two Thai *Leptospira* strains (Table 1). Average Nucleotide Identity (ANI) was calculated for each pair of *Leptospira* genomes by BLASTN analysis using JSpecies with the default setting [60].

To examine phylogenomic relationships and gain insights into the evolutionary history of *L. interrogans*, whole genome sequences for 97 *L. interrogans* strain were recruited for the analysis (Additional file 3: Table S2), using the genomic sequence of the closely related species *L. kirschneri* serovar Cynopteri strain 3522 CT (CYN) as an outgroup. The genomes were aligned, single nucleotide polymorphisms (SNPs) in conserved regions called, recombination sites filtered, and then concatenated as core-single nucleotide polymorphisms (core-SNPs) using parsnps in the Harvest package [61]. A phylogenetic tree constructed from aligned core-SNPs sequence was generated using the maximum-likelihood (ML) method with the GTR model and 1000 bootstrap replicates by RAxML [62]. The tree was edited and visualized using EvolView [63].

### Comparative genome analysis

The 97 *L. interrogans* genomes were subjected to pan-genome analysis using the Bacterial Pan Genome Analysis Pipeline (BPGA) package, version 1.2 [64]. The GenBank files obtained from the NCBI annotation were used as the input file. Orthologous proteins shared among the *L. interrogans* strains were clustered using USEARCH with 50% identity cut-off. Cluster of orthologous groups (COG) analysis was used to identify core genes, accessory genes and strain-specific genes. The pan-genome and core-genome were identified using exponential growth and decay models for each newly added *L. interrogans* genome using the power fit law equation “f(x) = a × x^b^”. A pan-genome curve was generated to examine whether the pan-genome of *L. interrogans* is open (having an unlimited gene repertoire) or closed (having a limited maximum number of genes in the gene pool) [65]. Moreover, strain-specific genes of strain CUDO5 and CUDO8 were identified using eggNOG [66] and the NCBI database, incorporating default parameters.

### Positive selection on orthologous genes in 13 *L. interrogans* genomes

To detect adaptive evolution in orthologous proteins shared among 13 representative *L. interrogans* strains, GET_HOMOLOUGUE was used to cluster protein-coding gene alignments with 80% identity and coverage [67]. Each of the 13 representative genome sequences was manually evaluated and converted to codon sequence alignment using PAL2NAL software, version 14.0 [68]. To detect positive selection, the rates of non-synonymous and synonymous substitutions were estimated using the site-model of the CODEML program in the PAML package: this determined the viable ratio of non-synonymous to synonymous substitution rates (*d*N/*d*S, or ω) at substitution sites among codon alignments [69]. Model M1a, that allowed sites in which ω =1 or < 1, was compared with model M2a that allowed sites with ω > 1. The Likelihood Ratio Test (LRT) and the likelihood statistic (2Δℓ) were calculated and compared with the critical value from the χ^2^ distribution with two degrees of freedom. If LRT rejected M1 and accepted M2 with an estimated ω > 1 this was interpreted as evidence of positive selection. The Bayes Empirical Bayes (BEB) was employed to identify positive selection sites by calculating posterior probability (PP) in each codon using a cut-off PP > 0.95 [70]. Phipack and GENECOV were used to identify genes under positive selection with recombination [71, 72]. Multiple testing corrections were undertaken using the procedure of Benjamini and Hochberg [73]: for all genes identified as being positive for selection, *q*-values were calculated from *p*-values using the QVALUE package from R [74], setting a false discovery rate (FDR) of 5% to control false positive detection. A binomial test was used to estimate associations between each of the COG and VFDB categories and the frequency of positive selection.

### Mapping of positive selection on protein tertiary structure

To construct protein tertiary structure, amino acid sequences deduced from genes under positive selection were modeled using homology-based protein modeling with the intensive mode in the Phyre2 (Protein Homology/analogy Recognition Engine) web server [75]. Three-dimensional (3D) protein structure was mapped with amino acid residues under positive selection and visualized by PyMOL (The PyMOL Molecular graphic System, Version 2.0 Schrödinger, LLC).

## Additional files

Additional file 1: **Tables S1-S9.** Genomic characterization of Thai *L. interrogans* serovar Paidjan strain CUDO5 and serovar Dadas strain CUDO8. (XLSX 158 kb)

Additional file 2: **Figure S1.** The structure of the *rfb* locus of *L. interrogans* serovar Paidjan strain CUDO5 and serovar Dadas strain CUDO8. (DOCX 4487 kb)

Additional file 3: **Table S1.** Average Nucleotide identity (ANI) of *Leptospira* strains and pan-genome analysis of 97 *L. interrogans* strains. (XLSX 26 kb)

Additional file 4: **Tables S1-S4.** Statistical analysis of positive selection in 13 representative *L. interrogans* strains. (XLSX 61 kb)

Additional file 5: **Figure S1.** Circular genome map of *L. interrogans* serovar Lai strain 56601 and the other 13 representative strains studied, with the location of 74 predicted genes under positive selection and recombination marked. (DOCX 1410 kb)

Additional file 6: **Tables S1-S2.** *Leptospira* strains used for serogroup and serovar typing. (XLSX 13 kb)

## Abbreviations

ANI: Average Nucleotide Identity
BEB: Bayes Empirical Bayes
COG: Cluster of Orthologous Group
CRISPR: Clustered regularly interspaced short palindromic repeat
EMJH: Ellinghuasen-McCullough-Johnson-Harris
LRT: Likelihood Ratio Test
mAb: Monoclonal antibody
pAb: Polyclonal antibody
SNP: Single Nucleotide Polymorphism
VFDB: Virulence Factor of Bacterial Pathogen Database
