# Correction to: Genomic analysis of *Leptospira interrogans* serovar Paidjan and Dadas isolates from carrier dogs and comparative genomic analysis to detect genes under positive selection

**DOI:** 10.1186/s12864-019-5612-6

**Published:** 2019-03-28

**Authors:** Alongkorn Kurilung, Chantisa Keeratipusana, Prapat Suriyaphol, David J. Hampson, Nuvee Prapasarakul

**Affiliations:** 1 Department of Microbiology, Faculty of Veterinary Science, Chulalongkorn University, Bangkok, Thailand; 2 Bioinformatics and Data Management for Research Unit, Office for Research and Development, Faculty of Medicine Siriraj Hospital, Mahidol University, Bangkok, Thailand; 3 Department of Infectious Diseases and Public Health, College of Veterinary Medicine and Life Sciences, City University of Hong Kong, Kowloon Tong, Hong Kong SAR; 4 Diagnosis and Monitoring of Animal Pathogens Research Unit, Department of Microbiology, Faculty of Veterinary Science, Chulalongkorn University, Bangkok, Thailand

**Correction to: BMC Genomics (2019) 20:168**

**https://doi.org/10.1186/s12864-019-5562-z**

Following the publication of this article [1], the authors noted an error in the caption of Fig. 4. The caption was published incorrectly as:

**Fig. 4:**
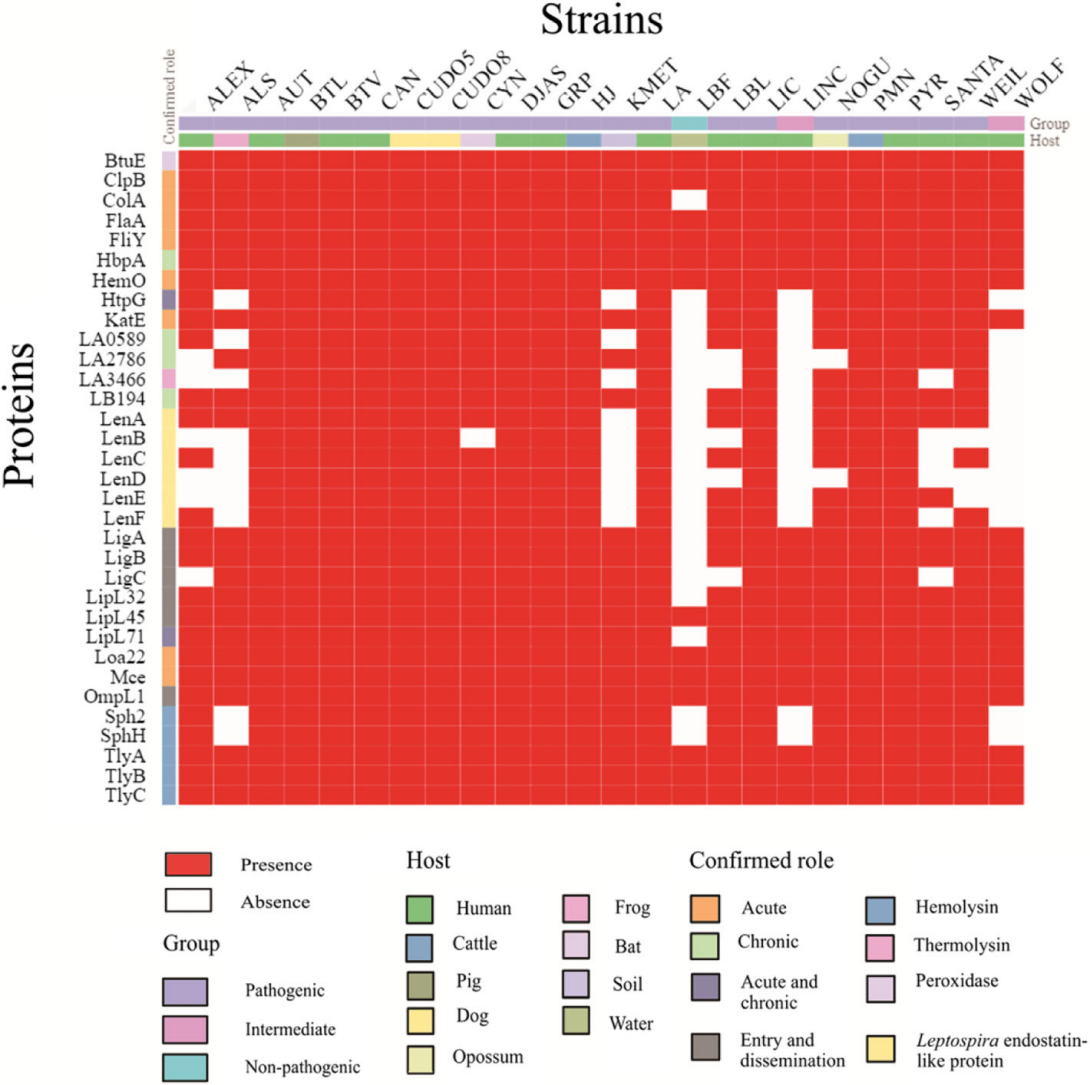
The distribution and conservation of 33 representative confirmed virulence genes in pathogenic, intermediate and non-pathogenic *Leptospira* species. Genes encoding for outer membrane protein (*loa22*), flagella motor switch protein (*fliY*), and hemolysins B and C (*tlyB* and *tlyC*) were conserved in all 24 *Leptospira* strains. Gene involved with chronic infection (*lb194*) was conserved only in pathogenic *Leptospira* strains. Strains CUDO5 and CUDO8 contained all of the 33 virulence genes

Figure 4 The distribution and conservation of 33 representative confirmed virulence genes in pathogenic, intermediate and non-pathogenic *Leptospira* species. Genes encoding for outer membrane protein (*loa22*), flagella motor switch protein (*fliY*), and hemolysins B and C (*tlyB* and *tlyC*) were conserved in all 24 *Leptospira* strains. Genes encoding was conserved only in pathogenic *Leptospira* strains. Strains CUDO5 and CUDO8 contained all of the 33 virulence genes

The correct figure and caption is reproduced in this Correction article:

The original article has been corrected.
