# Supplementary material for: Genomic analysis of Leptospira interrogans serovar Paidjan and Dadas isolates from carrier dogs and comparative genomic analysis to detect genes under positive selection
Source: BMC Genomics. 2019 Mar 4;20:168. doi: 10.1186/s12864-019-5562-z (PMC6399948; doi:10.1186/s12864-019-5562-z)
Supplement: Supplementary file 2 — Figure S1. The structure of the rfb locus of L. interrogans serovar Paidjan strain CUDO5 and serovar Dadas strain CUDO8. (DOCX 4487 kb) [file 12864_2019_5562_MOESM2_ESM.docx]

**
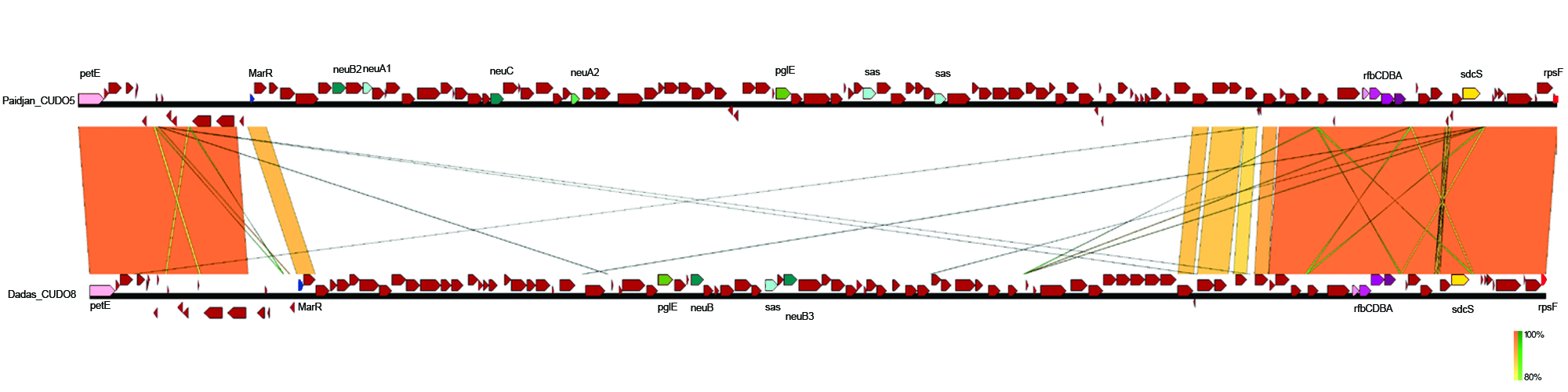
** **Additional file 2, Fig. 1 Comparison of sequence homology in *rfb* locus between *L. interrogans* serovar Paidjan strain CUDO5 and *L. interrogans* serovar Dadas strain CUDO8.** The CDSs are showed as directional frame boxes; the orange boxes between sequences indicate the percentage of sequence homology with the same directional CDSs and the green vertical boxes between the sequences represent the percentage of sequence homology with the invert directional CDSs (BLASTN identity 100% to 80%). Both serovars have conserved flanking genes upstream (*petE*) and downstream (*rpsF*) of the locus. Genes associated with rhamnose biosynthesis (*rfb ABCD*) and lipid A biosynthesis (*neuA1, neuB*) are found at the locus.
