# Supplementary material for: Genomic analysis of Leptospira interrogans serovar Paidjan and Dadas isolates from carrier dogs and comparative genomic analysis to detect genes under positive selection
Source: BMC Genomics. 2019 Mar 4;20:168. doi: 10.1186/s12864-019-5562-z (PMC6399948; doi:10.1186/s12864-019-5562-z)
Supplement: Supplementary file 5 — Figure S1. Circular genome map of L. interrogans serovar Lai strain 56601 and the other 13 representative strains studied, with the location of 74 predicted genes under positive selection and recombination marked. (DOCX 1410 kb) [file 12864_2019_5562_MOESM5_ESM.docx]

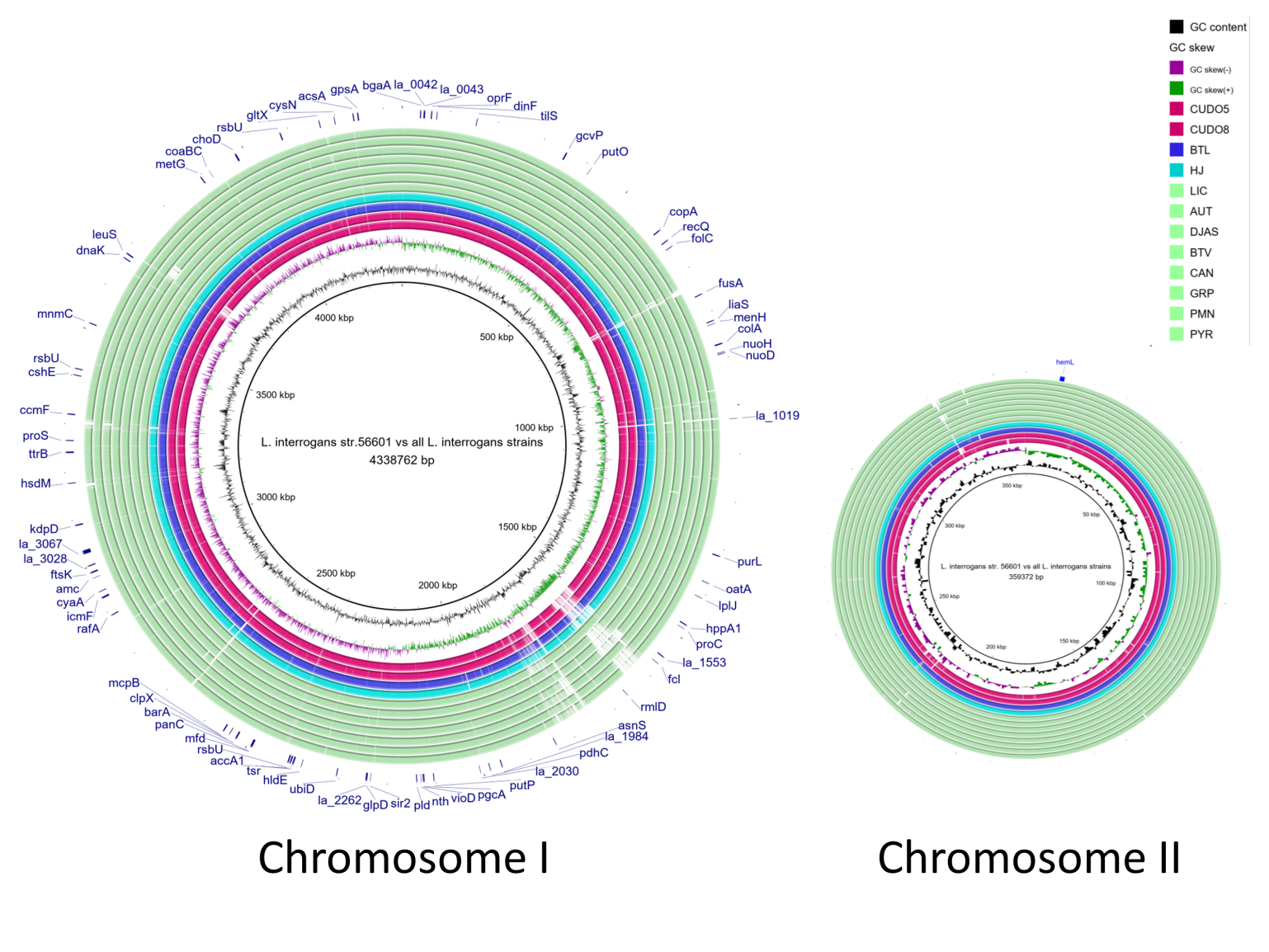


**Additional file 6, Fig 1.** **Circular genome map of *L. interrogans* serovar Lai strain 56601 and the other 13 representative strains studied, with the location of 74 predicted genes under positive selection and recombination marked.** Comparative sequence analysis was performed using *L. interrogans* strain 56601 as a reference (thick black innermost ring) against other representative related *L. interrogans* strains. The second inner ring indicates GC contents (black color) followed by the GC skew (purple and green colors). Each colored ring shows the host origin for each strain of *Leptospira* presented as follows: magenta for dogs, blue for pigs, cyan for cattle and light-green for humans. The outermost labels show the location of 74 predicted virulence genes under positive selection in *L. interrogans*. The intensity of the ring color represents the percentage of nucleotide identity from 50 to 90.
